# Black-blood dynamic contrast-enhanced carotid artery wall MRI with SRDIR preparation

**DOI:** 10.1186/1532-429X-15-S1-P246

**Published:** 2013-01-30

**Authors:** Zhaoyang Fan, Jingsi Xie, Yi He, Yutaka Natsuaki, Ning Jin, Daniel S Berman, Debiao Li

**Affiliations:** 1 Biomedical Imaging Research Institute, Cedars-Sinai Medical Center, Los Angeles, CA, USA; 2 Heart Institue and Imaging Center, Cedars-Sinai Medical Center, Los Angeles, CA, USA; 3 Radiology, Anzhen Hospital, Beijing, China; 4 Siemens Healthcare, Los Angeles, CA, USA; 5 Department of Bioengineering, University of California, Los Angeles, CA, USA

## Background

Inflammation plays a major role in atherosclerotic plaque progression and disruption [1]. Dynamic gadolinium contrast-enhanced (DCE) vessel wall imaging has been used to compute a set of contrast kinetic parameters that may characterize the extent of inflammation of carotid plaques [2-4]. However, previous DCE techniques are limited to a 2D bright-blood acquisition and thus the accuracy of wall signal assessment could be compromised. This work aimed to develop a 3D black-blood DCE technique.

## Methods

### Sequence

**Figure 1:**
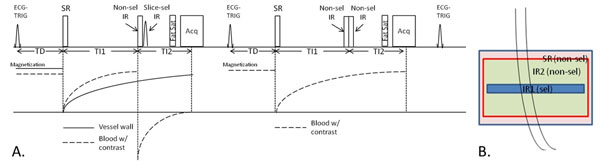
A. Sequence diagram of the SRDIR prepared RF spoiled GRE sequence. B. Locations of SR and DIR pulses.

An SRDIR (saturation recovery and double inversion recovery) preparation is combined with an RF spoiled gradient-echo sequence to achieve two aims: 1) To create T1-weighting for vessel wall; 2) To consistently null the blood with a fixed inversion time combination (TI1 and TI2) (Fig. 1). Bright-blood acquisition is interleaved with black-blood acquisition to enable arterial blood signal measurement as needed in kinetic modeling.

### Protocol

Nine healthy volunteers (7 F, 2 M; age 31-49 years) were scanned at 3T (Siemens Magnetom Verio) using a 4-channel bilateral carotid coil. The 3D DCE imaging using the SRDIR technique was conducted axially at the carotid bifurcations. Imaging parameters included: resolution = 0.6 × 0.6 × 2.0 mm^3^, 4 partitions, ECG triggering to minimize pulsation motion, 30 lines/RR, TI1/TI2 = 200/40 ms based on computer simulations. One-frame pre-contrast scan was followed by repetitive contrast-enhanced scans (40 s/frame, > 15 min), along with intravenous contrast (0.2 mmol/kg gadopentetate dimeglumine) injection and saline flush (30 ml) both at 0.2 ml/s. Through ROI analysis on the black-blood and bright-blood image series, respectively, the changes in signal intensity of carotid artery wall and lumen were obtained and used to compute the kinetic parameters (K^trans^, K_ep_, and V_p_) based on Toft's two compartmental model.

## Results

**Figure 2:**
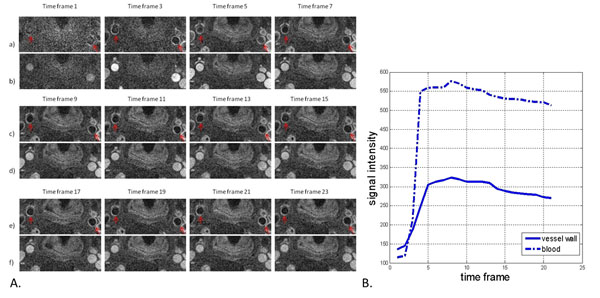
A. Interleaved black-blood (a, c, e) and bright-blood (b, d, f) images from one slice throughout the contrast injection process. B. Typical signal intensity vs. time curves of vessel wall and blood.

The luminal signal was consistently nulled regardless of the varying blood T1 values and carotid artery wall were clearly differentiated from the lumen on the DCE images (Fig. 2A). Signal intensity vs. time curves of carotid artery wall and blood were qualitatively similar to previous work (Fig. 2B). From the 9 subjects, K^trans^ = 0.062±0.027 min^-1^, K_ep_ = 0.664±0.334 min^-1^, and V_p_ = 39.24±8.34%.

## Conclusions

K^trans^ and V_p_ values obtained in this work were in accordance with previous studies. To our knowledge, this is the first work in which K_ep_ is investigated for carotid vessel wall. The healthy volunteer data indicates that SRDIR is a promising dynamic carotid vessel wall imaging technique. Clinical validations are currently underway.

## Funding

AHA11POST7650043

